# Retinoblastoma protein expression and its predictors in triple-negative breast cancer

**DOI:** 10.1038/s41523-020-0160-4

**Published:** 2020-06-05

**Authors:** Jaymin M. Patel, Andrew Goss, Judy E. Garber, Vanda Torous, Edward T. Richardson, Miriam J. Haviland, Michele R. Hacker, Gordon J. Freeman, Tessa Nalven, Brian Alexander, Larissa Lee, Laura C. Collins, Stuart J. Schnitt, Nadine Tung

**Affiliations:** 1 Division of Medical Oncology, Beth Israel Deaconess Medical Center, Boston, MA USA; 2 Harvard Medical School, Boston, MA USA; 3 Division of Medical Oncology, Dana-Farber/Brigham and Women’s Cancer Center, Boston, MA USA; 4 Department of Pathology, Massachusetts General Hospital, Boston, MA USA; 5 Department of Pathology, Brigham and Women’s Hospital, Boston, MA USA; 6 Department of Obstetrics & Gynecology, Beth Israel Deaconess Medical Center, Boston, MA USA; 7 Department of Epidemiology, Harvard T.H. Chan School of Public Health, Boston, MA USA; 8 Department of Radiation Oncology, Dana-Farber/Brigham and Women’s Cancer Center, Boston, MA USA; 9 Department of Pathology, Beth Israel Deaconess Medical Center, Boston, MA USA

**Keywords:** Breast cancer, Tumour biomarkers

## Abstract

Retinoblastoma protein (Rb) is a product of the *RB* tumor suppressor gene. Its expression is highly prevalent in luminal breast cancers and is critical to the success of cyclin-dependent kinase (CDK) 4/6 inhibitor therapy. Expression of Rb in triple-negative breast cancer (TNBC), tumors generally associated with basal biology, is not well known. However, heterogeneity among TNBC and presence of subtypes with luminal features are well described. The purpose of this study was to determine prevalence and predictors of Rb protein expression in *BRCA*1-associated and sporadic TNBCs. We studied 180 TNBC patients (70 *BRCA*1-associated and 110 sporadic). The clinical and pathologic features of these cases were previously assessed and reported. For this study, immunohistochemical stains for Rb were performed on tissue microarray sections. Details of treatment and outcome were abstracted from medical records. Fifty-one percent of TNBC were Rb positive (≥10% nuclei staining), and 85% of these cases had ≥50% nuclei staining. Rb expression was significantly associated with sporadic TNBC (71.4% vs 49.4%; *p* < 0.001), androgen receptor (AR) expression (16.5% vs 3.4%; *p* = 0.007), histologic grade 1 or 2 (9.9% vs 2.2%; *p* = 0.04), and first recurrence in bone (8.8% vs 1.1%; *p* = 0.03). Expression of p53 was not associated with Rb expression. Expression of Rb in TNBC was significantly associated with sporadic TNBC, AR expression, lower histologic grade, and metastasis to bone. These observations characterize a TNBC subtype with features suggestive of luminal-like biology and the potential to benefit from CDK 4/6 inhibition.

## Introduction

Triple-negative breast cancer (TNBC) is an aggressive subtype that comprises 15% of all breast cancers^1^. It is characterized by the absence of clinically meaningful estrogen receptor (ER), progesterone receptor, and human epidermal growth factor receptor (EGFR) 2 expression. Unfortunately, this classification fails to capture the underlying heterogeneity of TNBC observed at both clinical and molecular levels^2,3^. Among the major breast cancer subtypes, TNBC has the highest rate of relapse. An improved understanding of molecular subsets is essential to refine classification, identify new therapeutic targets, inform rational combinations for clinical testing, and, in turn, individualize patient management^4^.

Over the past 5 years, targeted inhibition of cyclin-dependent kinase (CDK) 4/6 in combination with endocrine therapy for hormone receptor-positive metastatic breast cancer has doubled progression-free survival when compared to endocrine therapy alone^5–7^. Intact retinoblastoma protein (Rb) is considered critical for success of this combination^8,9^. More than 90% of hormone receptor-positive (luminal-type) breast cancers have Rb intact^10^. While there is a rationale and preclinical data to support CDK 4/6 inhibition in a subset of TNBC, it is not clear which TNBCs are most likely to respond^11–13^.

Among the molecular subtypes of TNBC defined by gene expression profiling, the luminal androgen receptor group (LAR) is considered to be both molecularly and clinically the most similar to hormone receptor-positive breast cancers^14^. The LAR subtype demonstrates high expression of the androgen receptor (AR), as well as expression of luminal cytokeratins, and comprises 10–30% of all TNBC (ref. ^3^). In 2016, Tung et al. reported that certain features associated with hormone receptor-positive breast cancer, in particular lower histologic grade and older age, are predictive of AR expression in TNBC (ref. ^15^). It would be of great value to identify other features common to hormone receptor-positive breast cancer in TNBC, such as Rb expression, to help identify new therapeutic targets.

Preclinical data have demonstrated palbociclib-mediated cell cycle arrest in Rb-proficient TNBC cells and attenuation of this effect by Rb knockdown^13^. There are also in vitro and in vivo data on sensitivity to CDK 4/6 inhibition in LAR cells. These cells demonstrate significant differences in cell cycle dynamics, when compared to basal-like cells in single-cell analyses. Palbociclib-sensitive LAR cells exit mitosis and need CDK 4/6 activity to reenter the cell cycle. In contrast, palbociclib-resistant basal-like cells exit mitosis into a proliferative state independent of CDK 4/6 activity^11^.

Clinical trials evaluating CDK 4/6 inhibition in TNBC are currently underway^16^. Retrospective data on the frequency and predictors of Rb expression that may inform rational therapeutic combinations are needed. To address this, we assessed the frequency of Rb expression by immunohistochemistry (IHC) in a previously reported cohort of primary TNBC (ref. ^15^), and evaluated clinical and pathologic features predictive of Rb expression.

## Results

### Rb expression

Of the 180 TNBC, 51% were Rb positive (≥10% of nuclei staining for Rb); of those that were Rb positive, 85% had ≥50% of the nuclei staining. Among Rb-negative TNBC, 76% had <1% of nuclei staining.

### Clinical and pathologic features

Compared with patients that were Rb-negative, patients with Rb-positive TNBC were older (median age 48.0 vs 45.8 years), but this difference was not statistically significant (*p* = 0.18). Rb-positive TNBC were more likely to be histologic grade 1 or 2 than Rb-negative TNBC (9.9% vs 2.2%; *p* = 0.04). While there was more frequent nodal involvement and higher clinical stage among patients with Rb-positive compared with Rb-negative TNBC, these differences did not reach statistical significance (see Table 1). Clinical and pathologic features stratified by Rb nuclear staining scores 0–3 are shown in Supplementary Table 1.

**Table 1:** Clinical and pathologic features at presentation by retinoblastoma protein status.

	Retinoblastoma protein status		*P*
	Negative (<10%) *n* = 89	Positive (≥10%) *n* = 91	
Age at diagnosis (yrs)—mean ± SD	45.8 ± 9.9	48.0 ± 10.9	0.18
BRCA status—*n* (%)			<0.001
Carrier	44 (49.4)	26 (28.6)	
Sporadic	44 (49.4)	65 (71.4)	
Unknown	1 (1.1)	0 (0.0)	
Invasive histology—*n* (%)			0.87
Ductal	81 (91.0)	79 (86.8)	
Lobular	4 (4.5)	6 (6.6)	
Mixed	0 (0.0)	0 (0.0)	
Metaplastic	1 (1.2)	1 (1.1)	
Unknown	3 (3.4)	5 (5.5)	
Tumor size (cm)—median (IQR)^a^	1.9 (1.3, 2.8)	2.0 (1.5, 3.0)	0.23
Tumor grade—*n* (%)			0.04
1	0 (0.0)	1 (1.1)	
2	2 (2.2)	8 (8.8)	
3	87 (97.8)	79 (86.8)	
Unknown	0 (0.0)	3 (3.3)	
Lymphovascular invasion—*n* (%)			0.33
Present	28 (31.5)	36 (39.6)	
Absent	58 (65.2)	55 (60.4)	
Unknown	3 (3.4)	0 (0.0)	
Positive lymph nodes—*n* (%)			0.05
Present	13 (14.6)	30 (33.0)	
Absent	27 (30.3)	27 (29.7)	
Unknown	49 (55.1)	34 (37.4)	
T classification—*n* (%)			0.68
T1	54 (60.7)	47 (51.6)	
T2	24 (27.0)	31 (34.1)	
T3	4 (4.5)	3 (3.3)	
T4	2 (2.2)	2 (2.2)	
Unknown	5 (5.6)	8 (8.8)	
N classification—*n* (%)			0.09
N0	48 (53.9)	40 (44.0)	
N1	25 (28.1)	27 (29.7)	
N2	3 (3.4)	12 (13.2)	
N3	3 (3.4)	2 (2.2)	
Unknown	10 (11.2)	10 (11.0)	
AJCC stage—*n* (%)			0.06
1	28 (31.5)	18 (19.8)	
2	33 (37.1)	45 (49.5)	
3	9 (10.1)	15 (16.5)	
4	2 (2.2)	0 (0.0)	
Unknown	17 (19.1)	13 (14.3)	

*SD* standard deviation, *IQR* interquartile range, *AJCC* American Joint Committee on Cancer.

^a^Tumor size is missing for one woman in the retinoblastoma protein-negative group.

Rb expression in TNBC was significantly associated with the absence of a germline *BRCA*1 mutation and a higher likelihood of AR expression (both *p* ≤ 0.007)^15^. Of the 18 AR-positive (AR+) TNBC with available Rb data, 83.3% were Rb positive (see Table 2). No significant differences in luminal or basal cytokeratin staining or EGFR, programmed death-ligand 1 (PD-L1), or p53 IHC expression were noted according to Rb status (see Tables 2 and 3). Molecular features stratified by Rb nuclear staining scores 0–3 are shown in Supplementary Table 2.

**Table 2:** Molecular features at presentation by retinoblastoma protein status among the full cohort (*n* = 180).

	Retinoblastoma protein status		*P*
	Negative (<10%) *n* = 89	Positive (≥10%) *n* = 91	
Androgen receptor—*n* (%)			0.007
Negative/ weakly positive	84 (94.4)	74 (81.3)	
Positive (>10%)	3 (3.4)	15 (16.5)	
Unknown	2 (2.2)	2 (2.2)	
p53—*n* (%)			0.14
Negative	36 (40.4)	27 (29.7)	
Low positive (1 to <10%)	1 (1.1)	6 (6.6)	
Positive (≥10%)	45 (50.6)	52 (57.1)	
Unknown	7 (7.9)	6 (6.6)	
Epidermal growth factor receptor—*n* (%)			0.39
No staining	19 (21.4)	25 (27.5)	
≥10% positive	66 (74.2)	63 (69.2)	
Positive, unknown amount	1 (1.1)	0 (0.0)	
Unknown	3 (3.4)	3 (3.3)	
Cytokeratin 5/6—*n* (%)			0.49
No staining	31 (34.8)	37 (40.7)	
≥10% positive	55 (61.8)	52 (57.1)	
Positive, unknown amount	1 (1.1)	0 (0.0)	
Unknown	2 (2.3)	2 (2.2)	
Cytokeratin 14—*n* (%)			0.82
No staining	45 (50.6)	49 (53.9)	
≥10% positive	41 (46.1)	41 (45.1)	
Positive, unknown amount	1 (1.1)	0 (0.0)	
Unknown	2 (2.3)	1 (1.1)	
PD-L1 cancer—*n* (%)			0.73
Negative	63 (70.8)	68 (74.7)	
Positive (≥1%)	22 (24.7)	21 (23.1)	
Unknown	4 (4.5)	2 (2.2)

**Table 3:** Molecular features at presentation by retinoblastoma protein status among a subset (*n* = 51).

	Retinoblastoma protein status		*P*
	Negative (<10%) *n* = 29	Positive (≥10%) *n* = 22	
Cytokeratin 7/8—*n* (%)			0.16
Negative	1 (3.5)	0 (0.0)	
Equivocal/uninterpretable	6 (20.7)	1 (4.6)	
Positive	22 (75.9)	21 (95.5)	
Cytokeratin 18—*n* (%)			0.48
Negative	1 (3.5)	0 (0.0)	
Equivocal/uninterpretable	4 (13.8)	1 (4.6)	
Positive	24 (82.8)	21 (95.5)	
Cytokeratin 19—*n* (%)			1.0
Negative	1 (3.5)	1 (4.6)	
Equivocal/uninterpretable	1 (3.5)	0 (0.0)	
Positive	27 (93.1)	21 (95.5)	

### Treatment and outcomes

There were no significant differences in type of surgery, chemotherapy, or use of radiation between patients with and without Rb expression (see Table 4). Follow-up data was available for 79% of the patients; median follow-up was similar for Rb-negative (median: 12.1 years) and Rb-positive patients (median: 10.5 years; *p* = 0.10; see Table 5). Overall number of recurrences in this cohort is small limiting interpretation. No significant differences in either the frequency of local or distant recurrences were seen between Rb-positive and Rb-negative patients. However, site of first distant recurrence in bone was significantly more common among patients with Rb-positive (8.8%) than Rb-negative (1.1%) TNBC (*p* = 0.03). No significant difference in the incidence of brain metastasis was detected between the two groups.

**Table 4:** Treatment characteristics by retinoblastoma protein status.

	Retinoblastoma protein status		*P*
	Negative (<10%) *n* = 89	Positive (≥10%) *n* = 91	
Surgery—*n* (%)			0.56
Partial mastectomy	46 (51.7)	44 (48.4)	
Mastectomy	25 (28.1)	32 (35.2)	
Unknown	18 (20.2)	15 (16.5)	
Chemotherapy—*n* (%)	68 (76.4)	74 (81.3)	0.40
Anthracycline based	43 (63.2)	59 (79.7)	
Anthracycline + taxane	10 (14.7)	6 (8.1)	
Taxane based	0 (0.0)	1 (1.4)	
CMF	11 (16.2)	8 (10.8)	
Platinum based	1 (1.5)	0 (0.0)	
None	3 (4.4)	0 (0.0)	
Adjuvant radiation—*n* (%)			0.05
No	2 (2.2)	3 (3.3)	
Yes	38 (42.7)	54 (59.3)	
Unknown	49 (55.1)	34 (37.4)	

*CMF* cyclophosphamide methotrexate fluorouracil.

**Table 5:** Recurrence by retinoblastoma protein status.

	Retinoblastoma protein status		*P*
	Negative (<10%) *n* = 89	Positive (≥10%) *n* = 91	
Follow-up (years)—median (IQR)	12.1 (6.6, 15.9)	10.5 (3.2, 15.0)	0.10
Type of surgery—*n* (%)			0.56
Breast-conserving therapy	46 (51.7)	44 (48.4)	
Mastectomy	25 (28.1)	32 (35.2)	
Unknown	18 (20.2)	15 (16.5)	
Local recurrence—*n* (%)			0.41
Yes	9 (10.1)	13 (14.3)	
No	58 (65.2)	62 (68.1)	
Unknown	22 (24.7)	16 (17.6)	
Site of local recurrence—*n* (%)			
Chest wall	4 (4.5)	4 (4.4)	1.0
Scar	0 (0.0)	1 (1.1)	1.0
Ipsilateral breast	3 (3.4)	5 (5.5)	0.72
Lymph node	0 (0.0)	0 (0.0)	–
Distant recurrence—*n* (%)			0.22
Yes	15 (16.9)	24 (26.4)	
No	52 (58.4)	51 (56.0)	
Unknown	22 (24.7)	16 (17.6)	
Site of first distance recurrence—*n* (%)			
Bone	1 (1.1)	8 (8.8)	0.03
Lung	4 (4.5)	8 (8.8)	0.37
Liver	1 (1.1)	5 (5.5)	0.21
Brain	1 (1.1)	1 (1.1)	1.0
Nonlocal nodal group	0 (0.0)	1 (1.1)	1.0
Disseminated cutaneous	0 (0.0)	1 (1.1)	1.0
Overall incidence of brain metastasis	4 (4.5)	5 (5.5)	1.0

### Co-expression of Rb and AR by p53 status

As noted above, AR expression (≥10% cancer cells staining) was significantly associated with Rb expression (≥10% nuclear staining), whereas p53 expression (suggestive of a *TP53* mutation) did not significantly correlate with either AR or Rb staining (see Table 6). Contrary to expectation, p53 staining was more common among TNBC that expressed both AR and Rb staining than among patients that lacked both AR and Rb (71.4% vs 54.5%; *p* = 0.15); however, this difference did not reach statistical significance.

**Table 6:** Proportions of negative, low positive, and positive p53 by molecular features at presentation.

	p53 status			*P*
	Negative	Low positive	Positive	
Androgen receptor—*n* (%)				0.17
Negative/weakly positive (*n* = 146)	58 (39.7)	5 (3.4)	83 (56.8)	
Positive (>10%; *n* = 17)	4 (23.5)	1 (5.9)	12 (70.6)	
Unknown (*n* = 4)	1 (25.0)	1 (25.0)	2 (50.0)	
Retinoblastoma protein—*n* (%)				0.07
Negative (≤10%; *n* = 82)	36 (43.9)	1 (1.2)	45 (54.9)	
Positive (>10%; *n* = 85)	27 (31.8)	6 (7.1)	52 (61.2)	
Retinoblastoma protein and androgen receptor—*n* (%)				0.15
Both negative (≤10%; *n* = 77)	34 (44.2)	1 (1.3)	42 (54.5)	
Both positive (>10%; *n* = 14)	3 (21.4)	1 (7.1)	10 (71.4)	
Other (*n* = 72)	25 (34.7)	4 (5.6)	43 (59.7)	
Unknown (*n* = 4)	1 (25.0)	1 (25.0)	2 (50.0)	

## Discussion

In this study of 180 TNBC, 51% showed Rb expression by IHC. Lack of a *BRCA* mutation, lower tumor grade, and AR expression were all significantly associated with Rb expression. Lymph node involvement and older age were also more common among Rb-positive than Rb-negative TNBC, although these differences did not reach statistical significance. Having a first site of distant recurrence in bone also correlated with TNBC being Rb positive.

The features that we found associated with Rb expression in TNBC, i.e., older age, lower histologic grade, and a propensity for bone metastases are all frequently observed in hormone receptor-positive breast cancer, which is already known to have a relatively high propensity for Rb expression. Hormone receptor-positive breast cancer also has a high propensity for luminal features. The LAR subtype of TNBC, in addition to expressing luminal features, also is notable for AR overexpression. Our finding that AR expression in TNBC is significantly associated with Rb expression supports the notion of a more luminal subset of TNBC. Contrary to expectation, TNBC expressing both Rb and AR were more often p53 positive, a feature associated with aggressive TNBC, but findings did not reach statistical significance^17^. These clinical and molecular findings reflect previously described heterogeneity in TNBC, and may help to define a specific subset within TNBC that is more similar to hormone-positive/luminal-type breast cancer.

Our study is one of the few to describe frequency and predictors of Rb expression among TNBC. In 2009, Trere et al. prospectively evaluated Rb expression in 518 breast cancers, of which 53 were TNBC. They found Rb expression in 62.3% of TNBC, which they stratified further by phosphorylation status^10^. Comparison between that study and the current study is difficult due to their use of two different antibodies and different IHC scoring methods to define Rb positivity. Our cohort was enriched for *BRCA* mutant patients due to partial ascertainment from high-risk clinics and from an active trial program for patients with hereditary breast cancer. Given that Rb expression is less common among TNBC in *BRCA* mutation carriers, it is not surprising that the frequency of Rb expression was lower in our study (51% vs 62%, respectively).

Our findings have potentially important therapeutic implications. CDK 4/6 inhibitors, currently used only in hormone receptor-positive breast cancer, may also be beneficial for those TNBC patients that express Rb. Yamamoto et al. published in vitro and in vivo data, indicating palbociclib and the dual mammalian target of rapamycin kinase inhibitor MLN0128 showed synergistic activity in Rb+ models of ER-negative breast cancer^18^. Meanwhile, Tolaney et al. are conducting a clinical trial evaluating the role of abemaciclib in patients with Rb-positive TNBC that has been actively recruiting since May 2017, and will provide additional information on the potential role of CDK 4/6 inhibitors in Rb-positive TNBC (ref. ^16^).

Furthermore, our results demonstrate a statistically significant relationship between Rb expression and AR expression in TNBC. Clinical data supporting androgen blockade as a tolerable and potentially effective treatment option in a subset of TNBC patients continues to emerge^19,20^. However, which patients will benefit and what drug combinations are optimal is still unclear^21^. As with estrogen modulation in HR-positive metastatic breast cancer, androgen blockade may benefit from the simultaneous blockade of a cooperative signaling pathway, such as CDK 4/6–Rb (ref. ^22^). Liu et al. demonstrated in vitro activity of palbociclib in combination with enzalutamide in Rb-proficient and AR-positive TNBC cells^13^. More recently, Traina et al. reported the results from a phase II study of 118 AR-positive TNBC patients treated with enzalutamide 160 mg daily. Of the 78 patients deemed positive for AR expression at a ≥10% threshold, the clinical benefit at 16 weeks was 33%, median progression-free survival was 3.3 months, and median overall survival was 17.6 months (95%, CI, 11.6—not yet reached). Toxicity was minimal with fatigue as the only grade 3 symptom^19^.

Overall, targeting AR in TNBC has shown promise and combining this with CDK 4/6 inhibition would be a logical next step given its association with the Rb expression demonstrated in our study. Additional studies that explore the relationship of AR and Rb pathways in TNBC are needed. This has the potential to improve access to safe and tolerable FDA-approved oral medications for a greater percentage of breast cancer patients. However, evaluating this combination in a clinical trial setting may be challenging due to limitations in eligible patients. Our study, although not designed to determine prevalence, is notable for only 8% of TNBC (14/180) that co-express both Rb and AR.

It would also be valuable to assess PI3K status among Rb-positive TNBC given data that suggest enrichment of *PIK3CA* mutations in AR-positive TNBC (ref. ^23^). Data exist to support the combination of palbociclib with a PI3K inhibitor^24^. However, the clinical tolerability of this combination is unclear. In addition, expanded immune profiling that includes tumor mutation burden, albeit no correlation with PD-L1 staining in this study, would be of interest given emerging data on synergy of checkpoint inhibition with CDK 4/6 inhibition^25^. Lastly, whole-genome sequencing or whole-exome sequencing coupled with RNA sequencing of the 14 Rb+/AR+ samples will be useful to better understand and characterize this subset.

The appropriate level of Rb nuclear staining that is clinically meaningful or predicts response to CDK 4/6 inhibition remains an unanswered question. In our study, we chose to use ≥10% nuclear staining based on median staining intensity. However, the level of Rb expression by IHC staining that is clinically relevant will require prospective evaluation and validation. Furthermore, in the study by Traina et al., ≥10% AR nuclear expression by IHC had only modest positive predictive value for androgen blockade using enzalutamide. It is also unclear if AR expression using current IHC protocols is the best biomarker for predicting response to AR antagonists^19^.

Our study has certain limitations. In addition to its retrospective nature, selection bias for *BRCA1* mutations carriers generally enriched in the high-risk clinics used for recruitment in our study limits our ability to determine the actual prevalence of Rb among TNBC in the general population. Furthermore, it is not clear which antibody for evaluating Rb expression is best. Our pathologic assessment utilized a commercially available antibody that is among a number available with no clear data to delineate, which is optimal for clinical use or which predicts response to therapeutic agents.

In conclusion, we observed Rb expression in 51% of 180 TNBC. Clinical and pathologic evaluation revealed AR expression, lower histologic grade, a lack of a germline *BRCA* mutation, and the development of bone metastases as factors associated with Rb expression. These findings suggest clinical–pathologic similarities between a subtype of TNBC and hormone receptor-positive breast cancer. Our findings have important implications for use of CDK 4/6 inhibitors alone or in combination with AR blockade in a subset of TNBC.

## Methods

### Study population

The study population consists of 180 women diagnosed with stage I–III TNBC between 1989 and 2008. Eligible patients were identified in clinical databases from high-risk breast cancer clinics and an annotated Specialized Program of Research Excellence (SPORE) specimen bank at Beth Israel Deaconess Medical Center and Dana-Farber Cancer Institute.

### BRCA testing

As previously reported, *BRCA* status was determined either by heteroduplex testing of banked research blood samples or through commercially available CLIA-approved assays^15,26,27^.

### Clinical and pathologic data

Clinical data abstracted from medical records included age at diagnosis, clinical stage, tumor size, nodal involvement, germline *BRCA* status, treatment, and sites of recurrence. Pathologic features, including histologic type, Nottingham combined histologic grade, lymphovascular invasion, and lymph node involvement, were assessed on histologic sections of each tumor by the study pathologists (SJS and LCC).

### Immunohistochemical staining

IHC staining and scoring of tissue microarrays (TMAs) containing three 0.6 mm cores from each tumor was performed as previously described^15^ to assess expression of cytokeratins (CK 5/6, CK 14), EGFR, AR, PD-L1, and tumor suppressor protein p53 (TP53)^15^. A subset consisting of the first 51 cases were also evaluated for CK7/8, CK18, and CK19.

### Rb and p53 IHC staining

Rb staining was performed on TMA sections using a commercially available mouse monoclonal antibody to human Rb (clone G3-245, BD Biosciences, Franklin Lakes, NJ) and scored as 0 (<1% nuclear staining), 1 (≥1% to <10% nuclear staining), 2 (≥10% to <50% nuclear staining), or 3 (≥50% nuclear staining). Antibody clone G3-245 was raised against a Trp-E-Rb fusion protein and recognizes an epitope spanning amino acids 332–344 (DARLFDHDKTLQ) of human Rb (pp110–114 Rb). In addition to previously reported use and validation^28,29^, BD Pharmingen performed western blot analysis to demonstrate binding at various states of Rb phosphorylation (110–116 kD)^30^. Staining was performed in the Dana-Farber/Harvard Cancer Center Specialized Histopathology Core Laboratory, a CLIA-certified facility, using a protocol that is also used for routine standard-of-care diagnostics in the clinical immunohistochemistry laboratory within the Department of Pathology at Brigham and Women’s Hospital (BWH). All cases were run with a positive tumor control provided by the BWH Department of Pathology clinical laboratories, and with a tonsil control to ensure that the expected pattern of staining in normal proliferating cells (germinal center B cells and squamous epithelium) and other cell types found in reactive tonsil was observed. Although there is no consensus on what constitutes “Rb-positive” breast cancer, for our analysis, Rb-positive TNBC was defined as ≥10% nuclear staining. The highest staining observed among any of three TMA cores from each case determined its score. Similarly, p53 staining was performed on TMA sections using a mouse monoclonal antibody to human p53 (clone D0–1, ImmunoTech, Hostivař, Czech Republic) and scored as negative (<1% nuclear staining), low positive (≥1% to <10% nuclear staining), or high positive (≥10% nuclear staining).

### Statistical analysis

Study data were collected and stored in REDCap. All analyses were performed with SAS 9.4 (SAS institute, Cary, NC, USA). Descriptive data are reported as mean ± standard deviation, median (interquartile range), or proportion. Depending on distribution of data, continuous data were compared with a *t*-test or Wilcoxon rank-sum test, and categorical data were compared with *χ*^2^ or Fisher’s exact tests. All tests were two-sided and a *p* < 0.05 was considered statistically significant.

### Regulatory approval

This study was approved by Dana-Farber/Harvard Cancer Center Institutional Review Board and Scientific Review Committee. Given minimal risk to study patients, no informed consent was required.

## Supplementary information

Supplemental Table 1, 2

Reporting checklist

## Data Availability

The data generated and analyzed during this study are described in the following data record: 10.6084/m9.figshare.12174099 (ref. ^31^). The datasets supporting the findings of this study, are not publicly available due to restrictions in the IRB-approved protocol, and to protect research participant privacy. Data will be made available to authorized researchers who have submitted an IRB application. Please contact Dr. Nadine Tung (email address: ntung@bidmc.harvard.edu), for data access requests.
