# Supplementary material for: Retinoblastoma protein expression and its predictors in triple-negative breast cancer
Source: NPJ Breast Cancer. 2020 Jun 5;6:19. doi: 10.1038/s41523-020-0160-4 (PMC7275038; doi:10.1038/s41523-020-0160-4)
Supplement: Supplementary file 1 — Supplemental Table 1, 2 [file 41523_2020_160_MOESM1_ESM.pdf]

Supplemental Table 1

Clinical and pathologic features at presentation by retinoblastoma protein status

	Retinoblastoma Protein Status			
	**0 (<1%) n=68**	**1 (≥1% - <10%) n=21**	**2 (≥10% - <50%) n=14**	**3 (≥50%) n=77**
**Age at diagnosis (yrs)—mean ± SD**	45.7 ± 9.4	46.4 ± 11.4	49.6 ± 11.3	47.7 ± 10.9
**BRCA status—n (%)**				
**Carrier**	34 (50.0)	11 (52.4)	5 (35.7)	21 (27.3)
**Sporadic**	33 (49.5)	10 (47.6)	9 (64.3)	56 (72.7)
**Unknown**	1 (1.5)	0 (0.0)	0 (0.0)	0 (0.0)
**Invasive histology—n (%)**				
**Ductal**	60 (88.2)	21 (100.0)	13 (92.9)	66 (85.7)
**Lobular**	4 (5.9)	0 (0.0)	1 (7.1)	5 (6.5)
**Mixed**	0 (0.0)	0 (0.0)	0 (0.0)	1 (1.3)
**Metaplastic**	1 (1.5)	0 (0.0)	0 (0.0)	0 (0.0)
**Unknown**	3 (4.4)	0 (0.0)	0 (0.0)	5 (6.5)
**Tumor size (cm)—median (IQR)[1]**	1.9 (1.3-2.9)	1.7 (1.5-2.5)	2.3 (1.3-4.5)	2.0 (1.5-3.0)
**Tumor grade—n (%)**				
**1**	0 (0.0)	0 (0.0)	0 (0.0)	1 (1.3)
**2**	1 (1.5)	1 (4.8)	2 (14.3)	6 (7.8)
**3**	67 (98.5)	20 (95.2)	10 (71.4)	69 (89.6)
**Unknown**	0 (0.0)	0 (0.0)	2 (14.3)	1 (1.3)
**Lymphovascular invasion—n (%)**				
**Present**	21 (30.9)	7 (33.3)	4 (28.6)	32 (41.6)
**Absent**	45 (66.2)	13 (61.9)	10 (71.4)	45 (58.4)
**Unknown**	2 (2.9)	1 (4.8)	0 (0.0)	0 (0.0)
**Positive lymph nodes—n (%)**				
**Present**	10 (14.7)	3 (14.3)	6 (42.9)	24 (31.2)
**Absent**	21 (30.9)	6 (28.6)	2 (14.3)	25 (32.5)
**Unknown**	37 (54.4)	12 (57.1)	6 (42.9)	28 (36.4)
**T classification—n (%)**				
**T1**	40 (58.8)	14 (66.7)	7 (50.0)	40 (52.0)
**T2**	19 (27.9)	5 (23.8)	5 (35.7)	26 (33.8)
**T3**	4 (5.9)	0 (0.0)	0 (0.0)	3 (3.9)
**T4**	2 (2.9)	0 (0.0)	0 (0.0)	2 (2.6)
**Unknown**	3 (4.4)	2 (9.5)	2 (14.3)	6 (7.8)
**N classification—n (%)**				
**N0**	34 (50.0)	14 (66.7)	3 (21.4)	37 (48.1)
**N1**	21 (30.9)	4 (19.1)	7 (50.0)	20 (26.0)
**N2**	2 (2.9)	1 (4.8)	2 (14.4)	10 (13.0)
**N3**	2 (2.9)	1 (4.8)	0 (0.0)	2 (2.6)
**Unknown**	9 (13.2)	1 (4.8)	2 (14.3)	8 (10.4)
**AJCC Stage—n (%)**				
**1**	18 (26.5)	10 (47.6)	1 (7.1)	17 (22.1)
**2**	27 (29.7)	6 (28.6)	9 (64.3)	36 (46.8)
**3**	7 (10.3)	2 (9.5)	2 (14.3)	13 (16.9)
**4**	2 (2.9)	0 (0.0)	0 (0.0)	0 (0.0)
**Unknown**	14 (20.6)	3 (14.3)	2 (14.3)	11 (14.3)

Abbreviations: SD, standard deviation; IQR, interquartile range; AJCC, American Joint Committee on Cancer

[1]Tumor size is missing for one woman in the retinoblastoma protein <1% group

Supplemental Table 2
Molecular features at presentation by retinoblastoma protein status

	Retinoblastoma Protein Status			
	0 (<1%) n=68	1 (≥1% - <10%) n=21	2 (≥10% - <50%) n=14	3 (≥50%) n=77
**Androgen receptor—n (%)**				
Negative/ weakly positive	64 (94.1)	20 (95.2)	11 (78.6)	63 (81.8)
Positive (>10%)	2 (2.9)	1 (4.8)	3 (21.4)	12 (15.6)
Unknown	2 (2.9)	0 (0.0)	0 (0.0)	2 (2.6)
**p53—n (%)**				
Negative	27 (39.7)	9 (42.9)	7 (50.0)	20 (26.0)
Low positive	1 (1.5)	0 (0.0)	2 (14.9)	4 (5.2)
Positive	34 (50.0)	11 (52.4)	5 (35.7)	47 (61.0)
Unknown	6 (8.8)	1 (4.8)	0 (0.0)	6 (7.8)
**Epidermal growth factor receptor—n (%)**				
No staining	14 (20.6)	5 (23.8)	5 (35.7)	20 (26.0)
≥ 10% positive	52 (76.5)	14 (66.7)	8 (57.1)	55 (71.4)
Positive, unknown amount	0 (0.0)	1 (4.8)	0 (0.0)	0 (0.0)
Unknown	2 92.9)	1 (4.8)	1 (7.1)	2 (2.6)
**Cytokeratin 5/6—n (%)**				
No staining	21 (30.9)	10 (47.6)	6 (42.9)	31 (40.3)
≥ 10% positive	45 (66.2)	10 (47.6)	8 (57.1)	44 (57.1)
Positive, unknown amount	0 (0.0)	1 (4.8)	0 (0.0)	0 (0.0)
Unknown	2 (2.9)	0 (0.0)	0 (0.0)	2 (2.6)
**Cytokeratin 14—n (%)**				
No staining	33 (48.5)	12 (57.1)	9 (64.3)	40 (51.9)
≥ 10% positive	33 (48.5)	8 (38.1)	5 (35.7)	36 (46.8)
Positive, unknown amount	0 (0.0)	1 (4.8)	9 (64.3)	0 (0.0)
Unknown	2 (2.9)	0 (0.0)	0 (0.0)	1 (1.3)
**PD-L1 cancer—n (%)**				
Negative	49 (72.1)	14 (66.7)	12 (85.7)	56 (72.7)
Positive (≥1%)	16 (23.5)	6 (28.6)	2 (14.3)	19 (24.7)
Unknown	3 (4.4)	1 (4.8)	0 (0.0)	2 (2.6)
